# Native Right Ventricular Outflow Tract Stenting in a Child with Tetralogy of Fallot and Absent Left Pulmonary Artery

**Published:** 2013-11-30

**Authors:** Akbar Molaei, Mahmud Meraji, Elaheh Malakan Rad

**Affiliations:** 1 Rajaei Cardiovascular & Medical Research Center, Pediatric Cardiology Department, Iran University of Medical Sciences; 2 Children’s Medical Center, Tehran University of Medical Sciences, Tehran, Iran

**Keywords:** Stenting, Tetralogy of Fallot, RVOT Stenosis

Although percutaneous placement of intravascular stents in congenital heart disease is a common practice, there are few reports regarding native right ventricular outflow tract (RVOT) stenting in children^[^^1^^]^. Stenting of conduit stenoses are more commonly reported^[^^2^^]^.

 In the preoperative setting, palliation of significant cyanosis by balloon valvuloplasty or RVOT stenting has been advocated by some as a means for reducing symptomatic cyanosis in patients with severe annular hypoplasia. Improvement in antegrade flow is thought to simultaneously enhance pulmonary arterial growth by augmenting pulmonary blood flow. 

 Most of transcatheter interventions for relieving RVOT were done for conduit stenosis. There are few reports about native RVOT stenting, and to the best of our knowledge there are very few reports on native RVOT stenting in tetralogy of fallot (TOF) with absent left pulmonary artery^[^^3^^]^.

 A 9-year-old child was admitted with cyanosis noted from birth with failure to thrive, cyanotic spells and worsening cyanosis. The patient had undergone central modified Blalock- Tausig (MBT) shunt and right MBT shunt at the ages of three and six respectively. At this admission the child weighed 17 kg, had severe systemic desaturation (<55%) and severe cyanosis, digital clubbing and a New York Heart Association (NYHA) classification of class IV. Clinical examination revealed unremarkable pulmonary examination and 3/6 systolic heart murmur at pulmonary focus. 

**Fig. 1:**
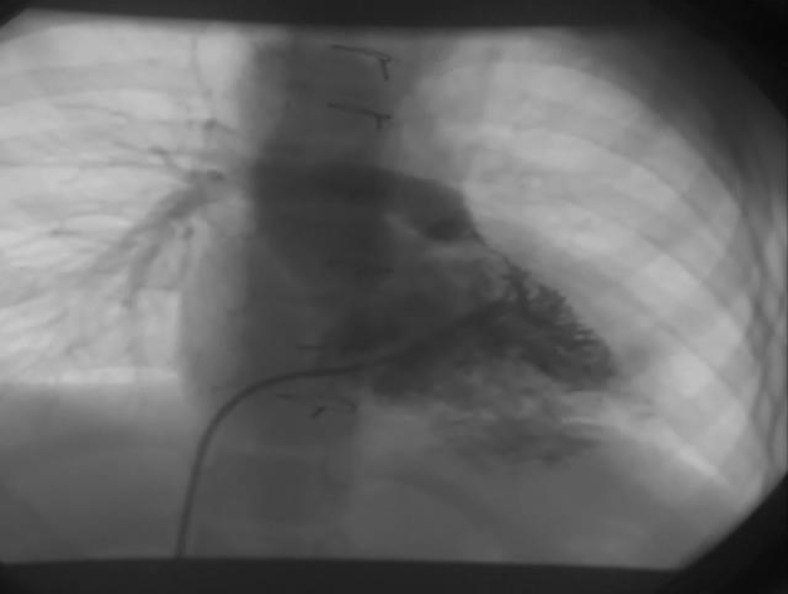
Right ventricular injection in anteroposterior view shows severe right ventricular outflow tract stenosis and absent left pulmonary artery

**Fig. 2:**
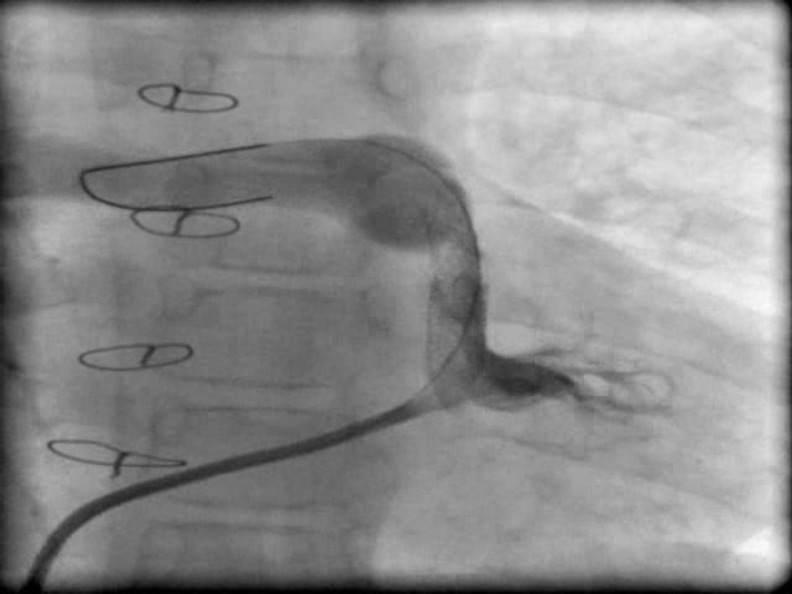
Right ventricular injection in anteroposterior view after right ventricular outflow tract stenting shows significant resolved stenosis and increased right lung blood flow without pulmonary valve involvement.

 EKG revealed normal sinus rhythm, right axis deviation and severe right ventricular hypertrophy (RVH). Echocardiography showed TOF anatomy, severe RVOT stenosis with 75 mmHg pressure gradient, RVH, abscent left pulmonary artery branch (LPA), non-functioning previous MBT shunts and major collateral arteries originating from descending aorta. On catheterization, both previous MBT shunts were occluded, the right pulmonary artery (RPA) was small with fairly acceptable arborization. Left lung was supplied by major collateral arteries originating from descending aorta and severe RVOT stenosis was present (Fig. 1). RVOT stenting was performed by two consecutive stents (17×7 mm Express LD, Boston Scientific. USA and 18×5 mm Racer renal stents, Medtronic USA). Post stenting angiography showed significant increase in pulmonary blood flow to the right lung (Fig. 2).

 The arterial oxygen saturation rose to 83%. At six-month follow-up, his arterial oxygen saturation was maintained at above 80% and NYHA functional class was improved to class II. Palliative procedures for relieving RVOT stenosis include either surgical or transcatheter interventions. The indications for RVOT stenting are RV-to-PA conduit stenosis, residual infundibular stenosis after intracardiac repair, TOF with hypoplastic branch pulmonary arteries after palliative shunt surgery, pulmonary atresia after perforation of atretic segment, and RV hypertrophic cardiomyopathy and also as a bridge to surgery. RVOT stenting is also usually indicated in cases in those patients who are not amenable to total surgical repair. Our patient had absent LPA, small RPA and poor clinical condition. Thus, we carried out percutaneous RVOT stenting.

RVOT stenting in such patients theoretically can lead to two major problems: overflow and edema of the right lung with resultant vasculopathy in the long term, and free pulmonary valve regurgitation with its consequences that include right ventricular dilatation and dysfunction.

 In our case, none of these complications occurred, because we chose the stent size with scrutiny and we did not sacrifice the pulmonary valve. Also we did not have complications such as stent migration, ventricular arrhythmias, collapse or fracture of the stent and recurrent stenosis during follow up. To our knowledge the patient has not undergone any curative operation by now.

 RVOT stenting provides an effective palliative modality for children with TOF and unfavorable pulmonary artery anatomy. Although re-stenosis can occur but it responds to re-dilatation.

 In high risk patients such as our patient with severe cyanosis and high hemoglobin level and blood viscosity, the RVOT stenting decreases perioperative morbidity and mortality^[^^4^^]^. In conclusion native RVOT stenting is an effective and safe procedure in appropriately selected patients, especially in whom total correction is not possible. This procedure causes better growth of pulmonary artery branches, decreases right ventricular hypertrophy and increases left ventricular volume. Although many of these stents cannot be dilated to adult size, their efficacy in small infants and children in whom further surgery will ultimately be required is remarkable.
